# Improvement in diagnostic-therapeutic care pathways for women with migraine: an Italian Delphi panel

**DOI:** 10.3389/fneur.2024.1436258

**Published:** 2024-09-05

**Authors:** Sabina Cevoli, Piero Barbanti, Cinzia Finocchi, Laura Benedan, Paolo Mariani, Nicoletta Orthmann, Salvatore Bauleo, Paola Brusa, Dorella Cianci, Luca Marozio, Sara Masseroni, Roberto Sangermani, Fabio Frediani, Gianni Allais

**Affiliations:** ^1^Programma Cefalee e Algie Facciali, IRCCS Istituto delle Scienze Neurologiche di Bologna, Bologna, Italy; ^2^Headache and Pain Unit, IRCCS San Raffaele, Rome, Italy; ^3^San Raffaele University, Rome, Italy; ^4^SC Neurologia PO Levante, Ospedale San Paolo, ASL 2 Savonese, Savona, Italy; ^5^Department of Economics, Management and Statistics, University of Milano-Bicocca, Milan, Italy; ^6^Fondazione Onda, Osservatorio nazionale sulla salute della donna e di genere ETS, Milan, Italy; ^7^Medicina Generale, Casa della Salute di Zola Predosa, AUSL, Bologna, Italy; ^8^Department of Scienza e Tecnologia del Farmaco, University of Turin, Turin, Italy; ^9^Università Lumsa Roma, Rome, Italy; ^10^Department of Surgical Sciences, Obstetrics and Gynecology 1, University of Turin, Turin, Italy; ^11^SC Oncologia, ASST Santi Paolo e Carlo, Milan, Italy; ^12^Freelance Pediatric Medicine Specialist, Milan, Italy; ^13^Headache Center, Neurology and Stroke Unit, S. Carlo Hospital, ASST Santi Paolo Carlo, Milan, Italy; ^14^Department of Surgical Sciences, Women’s Headache Center, University of Turin, Turin, Italy

**Keywords:** migraine, woman, Delphi, gynecologist, neurologist, pediatrician, oncologist, pharmacist

## Abstract

**Background:**

Migraine is a highly underestimated and burdensome disease. Real-world studies evidence that migraine is more frequent and severe in women than men. However, to this day, no diagnostic-therapeutic pathways exist to satisfy the specific needs of female patients.

**Methods:**

In this study, migraine experts, specialists in women’s health, patient, and decision makers, analyzed the diagnostic and therapeutic options for women with migraine across various ages and health conditions within the Italian healthcare system. A Delphi approach was used to formulate statements and achieve a consensus.

**Results:**

Gaps in clinical practice were identified, and strategies to accommodate women’s needs were proposed. The experts agreed that a socio-behavioral intervention should be planned before any pharmacological treatment in pediatric/adolescent female patients and that the assessment of migraine with aura is considered crucial for adult women requiring contraceptive therapy. Acupuncture emerged as an effective treatment for pregnant and breastfeeding women, and hormone-replacement therapy selection in menopausal patients requires careful consideration to mitigate safety risks. The experts highlighted the absence of literature and guidelines for the management of migraine in women undergoing assisted reproductive procedures or oncological treatment. In light of these observations, the experts advocated the establishment of multidisciplinary collaborations between neurologists/headache specialists and other healthcare professionals, including general practitioners, pediatricians, gynecologists, and oncologists. Comprehensive migraine education for all healthcare professionals potentially involved in managing the disease, including pharmacists, was emphasized. Efforts to increase migraine awareness among women should be prioritized.

**Conclusion:**

The insights gained from this Italian consensus study should serve to develop an improved, female-specific pathway to diagnose and treat migraine.

## 1 Introduction

Migraine is a common neurologic disease usually characterized by a unilateral and pulsating headache, accompanied by other bothersome symptoms (e.g., nausea, photophobia, phonophobia). The inter- and intra-individual clinical manifestations of migraine can be extremely variable, hence its diagnosis is supported by the International Classification of Headache Disorders in their third edition (ICHD-3) (1). The current diagnostic criteria provide guidelines to distinguish migraine from other primary headache disorders and discriminate their different types (without aura, with aura, and chronic) and their subtypes (2). A family history of migraine is a risk factor for developing the disease and should be assessed thoroughly during the diagnostic process (3). Detailed recommendations on the use of preventive and acute migraine treatments, spanning from lifestyle modification to pharmacological therapies, are also available (4).

Currently, migraine is ranked among the most debilitating conditions worldwide. Recent estimates revealed that the prevalence of global migraine has reached up to 14–15% and accounts for a total of 45.1 million years lived with disability (5–8). Not surprisingly, the economic burden of migraine on patients, healthcare system and society is equally substantial (9–11), with an average annual cost per patient of 1,222 euros calculated across eight European countries (12).

The incidence of migraine varies throughout life, peaking at 35 and 50 years of age and subsequently declining at older ages (13, 14). After puberty, its occurrence shows a net contrast between genders, being approximately three times more frequent in females than males (14, 15). Migraine is not only more common but also more severe in females, with migraine-associated symptoms (e.g., nausea, phonophobia) being significantly more frequent among females than males (16). On average, women experience prolonged migraine episodes, with perimenstrual and non-perimenstrual migraine being 1.62 and 1.15 times longer than migraine experienced by males, respectively (17). Indeed, women usually require a longer time to recover from migraine attacks than men, are more likely to visit emergency or urgent care units for any headache pain, and use of anti-migraine drugs. According to the Migraine Disability Assessment Questionnaire (MIDAS), the headache-related disability caused by migraine is greater in females than in males; this result has negative implications on many aspects of life, including reduction of work productivity, inability to perform housekeeping tasks, and withdrawal from social activities (18, 19). Furthermore, migraine’s negative effects on neurocognitive functions are more pronounced in females compared to males (20).

Several aspects help to explain the observed gender difference in migraine prevalence and impact. Fluctuating estrogen levels are likely to play a key role in the pathogenesis of female migraine; the presence of hormone receptors in specific brain regions suggests that estrogen variations affect neural circuits and modulate brain function and structure. Genetic polymorphisms or mutations may also contribute to the observed migraine sex disparity, along with stress, behavioral and nutritional factors (13, 14).

Despite the disproportionate disease burden, women with migraine may struggle to receive appropriate medical assistance. The higher prevalence of migraine in women has contributed to its classification as a “female disorder,” often perceived as less serious by healthcare providers. This gender bias can result in women being stigmatized and receiving less comprehensive care, potentially leading to underdiagnosis, undertreatment, and medication overuse headache (21, 22). In addition, pregnancy and breastfeeding limit access to many existing migraine medications considered unsafe during these critical periods, leaving women with persistent headaches and limited therapeutic options (23, 24).

The worse clinical condition of women with migraine compared to men was observed and confirmed in Italy. When the socio-economic status was examined, women had a higher number of lost days at work and participated to fewer social activities than men, although results were not statistically significant. Women were also more likely to come in to work despite the presence of migraine. Not surprisingly, results from the Migraine-Specific Questionnaire (MSQ) indicated that women’s quality of life was considerably lower than men’s, mainly due to disruption of daily activities (25).

Despite the well-recognized burden of the disease, migraine still appears to be widely underestimated. A study conducted across ten European countries showed that only low percentages of all individuals with migraine consult either a general practitioner (GP) (ranging from 9.5 to 18.0%) or a specialist (ranging from 3.1 to 15.0%) (26). In Italy, the prevalence of migraine and the rate of its diagnosis and treatment appear to be even more dramatic compared to other European countries, with a prevalence of 11.6% (27), with peaks of 24.7% (28) and 42.9% (29). Data from the Eurolight Study showed that only 15.8% of Italian patients with migraine consulted either the GP (9.5%) or a specialist (6.3%) and, among eligible patients, only 1.6% were treated with preventive drugs (26). According to a study conducted among 2,675 patients from 10 Italian headache centers, only 26.8% previously received a correct diagnosis and the large majority (82.8%) used non-specific drugs to treat migraine attacks (30). Of all the Italian patients presenting chronic migraine, a mere 52.6% was seen by a specialist, and the average number of specialists consulted by each patient was 7 (31). These figures show a great deal of uncertainty for migraine patients on who to consult and where to obtain the appropriate medical attention.

Overall, the need to raise awareness about migraine and improve the current diagnostic-therapeutic pathway of patients is paramount. Moreover, in women with migraine, particular needs for a complex and multidisciplinary approach emerge during specific timepoints of their reproductive life or upon development of comorbidities. Given that this disease predominantly affects the female gender in terms of both frequency and severity, *ad hoc* pathways should be created for women, through which female patients are effectively treated by a multidisciplinary team according to their age category, health condition and/or any other female-related aspect emerging throughout their lives. Through a Delphi consensus, a panel of experts analyzed the actual literature on female migraine management and existing diagnostic-therapeutic care pathways that female patients may follow in the Italian healthcare setting, identifying both the strengths and pitfalls. The results are discussed in this study and aim to pave the way for the development of an improved and standardized approach, tailored to meet the unique needs of female patients worldwide, to diagnose and treat migraine.

## 2 Methods

### 2.1 The Delphi panel

Consensus methods are an effective strategy for involving a diverse range of stakeholders, including researchers, clinicians, patients, and policymakers, to collect distinct perspectives and expertise. By fostering collaboration and encouraging shared decision-making, these methods play a crucial role in guiding evidence-based clinical research and practice. The Delphi method stands out as a widely utilized consensus approach in clinical research, particularly when addressing intricate or uncertain subjects. Its fundamental components comprise anonymity, iterative processes, controlled feedback, and the statistical reliability of consensus (32). Through multiple rounds of anonymous surveys or questionnaires, experts can offer feedback and refine their responses based on the collective insights of the group. This iterative approach facilitates the attainment of consensus among experts, leading to well-informed decisions and recommendations within the realm of clinical studies (33).

Due to its efficacy in fostering collaboration and gathering expert opinions, the Delphi method was chosen as the preferred approach to solicit insights from a distinguished panel of experts specializing in migraine research and women’s health within Italy. Employing this method, the study aimed to delve into the current diagnostic protocols and clinical management options for women afflicted by migraine in the Italian healthcare system. By leveraging the expertise of the Delphi panel, the research sought to uncover valuable insights to guide future strategies to improve migraine management practices in Italy.

**Figure 1:**
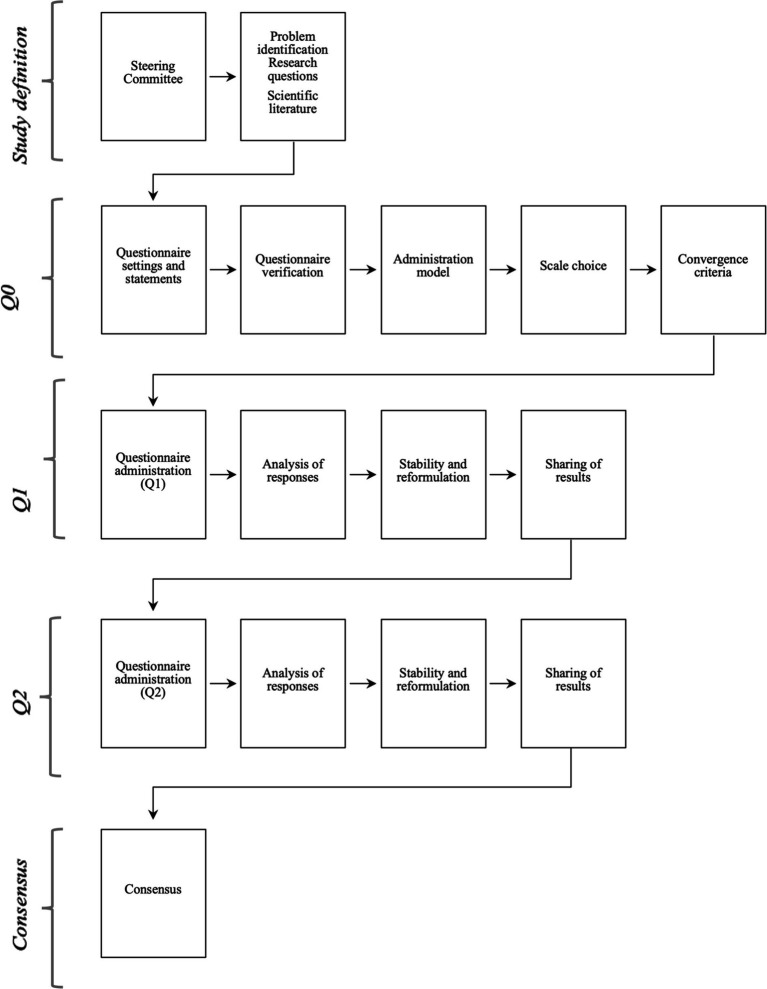
Flow-chart of the methodological process followed in the Delphi.

We followed the standards described for reporting both the methodologies and outcomes of Delphi studies, as outlined by Diamond et al. (34) and Boulkedid et al. (35). In our study, steering committee members were allowed to participate as panellists due to their deep expertise and unique insights. This practice is found in the literature (36). Figure 1 depicts the comprehensive flowchart describing the methodological process employed throughout this study.

### 2.2 Expert panellists and questionnaire development

The steering committee was composed by five neurologists/headache specialists affiliated with the Italian Neurological Association for Headache Research (ANIRCEF), with proven expertise (years of activity, publication record) in the management of migraine. Two methodologists supported the steering committee in structuring the discussion and preparation/organization of the statements for the Delphi questionnaire, but they were not involved in drafting the clinical content of the statements. The relevant topics and the clinical questions were identified during two preparatory virtual meetings. During the first meeting the discussion focused on the identification of the specific settings and needs of women with migraine. Between the two meetings, a literature search was performed to identify the existing background and the potential gaps in knowledge according to the defined settings. Following an in-depth literature review and drawing upon their clinical expertise, the committee pinpointed essential topics for analysis, extensively discussed during the second meeting. Subsequently, the experts formulated and validated the corresponding statements/questions through a series of offline meetings between June and October 2023. The statements were then integrated into an online questionnaire. To achieve consensus on the statements participants were tasked with providing ratings on a 4-point Likert scale, ranging from 1 (indicating strong disagreement) to 4 (indicating full agreement). The ratings were defined as follows: 1 = “Fully Disagree,” 2 = “Partially Disagree,” 3 = “Partially Agree,” 4 = “Fully Agree.” The omission of a neutral option was aimed at prompting respondents to take a clear position in terms of agreement or disagreement, albeit with varying degrees.

*A priori* criteria were established for defining consensus achievement, with a minimum agreement threshold set at 67% (34). Besides, some questions concerning the current general management of migraine and the desirable characteristics of the optimal path for the female patient were also included. For those questions, no threshold was defined, as they were meant to gauge the current opinion on the situation. The questionnaire was submitted to a panel of 12 experts, selected according to their expertise in the management of migraine, their specific field of knowledge (gynecology, oncology, pediatrics, general medicine, pharmacology, gender medicine), their willing in responding to the questionnaire, their previous participation and interest in activities aimed to increase awareness about the disease. The questionnaire was submitted through an online platform during November 2023 (first round) and December 2023 (second round). Answers were collected within 1 week.

### 2.3 Data analysis

The results are expressed as percentages of agreement for all the statements and percentage of responses to the questions. In addition, written explanations to some questions were provided anonymously by the expert panellists. Responses were mandatory for all the questions.

## 3 Results

### 3.1 Panellists

The panel evaluating the questionnaire was composed of 12 members, with different expertise, with the majority (33%) belonging to neurology. Other specialties included general medicine, pediatrics, gynecology, oncology, an expert in clinical pharmacology and pharmaceutical regulations, a socio-pedagogical researcher, a headache patient, and an expert in communication and migraine.

### 3.2 Delphi first round

Eighty-one items were evaluated by the panellists through a web-based survey. Of those, 24 were questions investigating either the current situation of the journey of a female migraine patient, or the best practice for optimal management, and 57 were statements to be evaluated with a Likert scale. Fifty-three statements reached an agreement at the first round with only four statements not reaching the consensus. The complete text of the statements/questions proposed in the first round and the respective percentages reached are shown in Table 1.

**Table 1:** First round survey full text and results.

1. General framework, perception of the illness: indicate the degree of agreement with the following statement		
		Agreement
In your experience, the woman who suffers from migraine	1.1 Is aware that migraine is a pathology	58%
	1.2 Is aware of having a pathology but does not know whom to turn to	83%
	1.3 Is subjected to a high burden of the disease in social terms (low quality of life, missed participation in social activities, negative impact on relationships, need for support)	83%
	1.4 Is subjected to a high burden of the disease in terms of work/economics (high percentage of absenteeism, negative impact on career prospects)	83%
	1.5 Perceives her symptoms as a stigma	92%

2. Diagnosis of the condition: please answer to the following questions					
		<1 year	Between 1 and 3 years	Between 3 and 5 years	>5 years
In your experience	2.1 On average, how long between the onset of symptoms and the first medical assessment?	8%	33%	25%	33%
	2.2 On average, how long between the first medical assessment and the diagnosis?	17%	42%	8%	33%
		<1 month	Between 1 and 6 months	Between 6 months and 1 year	Over 1 year
	2.3 What is the average waiting time for the first specialized neurological visit at a headache center or with an expert neurologist?	–	50%	42%	8%

3. General features of current management: Indicate the level of agreement with the following statement		
		Agreement
In your experience, the current management of patients with migraine	3.1 It is insufficient	83%
	3.2 It is characterized by the lack of multidisciplinary approach	100%
	3.3 It is characterized by the lack of continuity of care	92%
	3.4 It is characterized by the lack of medical references at the territorial level	100%
	3.5 It is heterogeneous and non-standardized in Italy	100%

3. General features of current management: Indicate, for each stage of life, the responses you consider most appropriate							
In your experience, the primary point of reference for a woman experiencing migraine symptoms in various stages of life and specific health conditions is represented by (possible more than one answer):							
	Pediatric age	Adolescence	Adulthood with menstrual or menstruation-related migraine	Adulthood while undergoing contraceptive therapy	Adulthood, pregnancy and breastfeeding	Menopause	Adulthood during oncological treatment
3.6 The pharmacist	17%	25%	33%	17%	8%	33%	0%
3.7 The psychologist	0%	8%	0%	0%	0%	0%	0%
3.8 The pediatrician	92%	33%	0%	0%	8%	0%	0%
3.9 The general practitioner	8%	75%	50%	50%	8%	33%	58%
3.10 The child neuropsychiatrist	17%	8%	8%	0%	0%	0%	0%
3.11 The neurologist and/or headache specialist	8%	25%	25%	25%	25%	50%	25%
3.12 The gynecologist	0%	8%	92%	100%	92%	50%	0%
3.13 The oncologist	0%	0%	0%	0%	0%	0%	92%
3.14 Other	0%	0%	0%	0%	8%	0%	0%

4. Desirable management: Indicate, for each stage of life, the responses you consider most appropriate							
In your experience, the primary point of reference for a woman experiencing migraine symptoms in various stages of life and specific health conditions should be represented (possible more than one answer):							
	Pediatric age	Adolescence	Adulthood with menstrual or menstruation-related migraine	Adulthood while undergoing contraceptive therapy	Adulthood, pregnancy and breastfeeding	Menopause	Adulthood during oncological treatment
4.1 The pharmacist	0%	0%	8%	8%	8%	17%	8%
4.2 The psychologist	8%	17%	0%	0%	0%	0%	0%
4.3 The pediatrician	83%	25%	0%	0%	8%	0%	0%
4.4 The general practitioner	8%	75%	67%	50%	50%	50%	58%
4.5 The child neuropsychiatrist	42%	25%	0%	0%	0%	0%	0%
4.6 The neurologist and/or headache specialist	33%	50%	67%	58%	58%	67%	58%
4.7 The gynecologist	0%	0%	58%	100%	83%	83%	0%
4.8 The oncologist	0%	0%	0%	0%	0%	0%	92%
4.9 Other	0%	0%	0%	0%	0%	0%	0%

5. Considering a patient in pediatric and adolescent age: Indicate the level of agreement with the following statement		
		Agreement
In the management of migraine in pediatric and adolescent patients	5.1 It is crucial to investigate the social aspects that may trigger migraine attacks, especially during the transition from pre- to post-puberty	100%
	5.2 The first intervention is socio-behavioral to prevent episodes and/or reduce their frequency	92%
	5.3 A combined intervention (socio-behavioral and pharmacological) is necessary if the socio-behavioral intervention alone has not yielded improvements	100%
	5.4 The psychologist is involved only if conditions of stress or anxiety are hypothesized	75%
	5.5 The psychiatrist is involved only if a psychopathological disorder is hypothesized	100%
	5.6 It is necessary to increase awareness among parents regarding the pathology and migraine symptoms	100%

6. Considering an adult patient with menstrual migraine: Indicate the level of agreement with the following statement		
		Agreement
In the management of adult patients with menstrual migraine or menstruation-related migraine	6.1 Specialized consultation is necessary only in the case of prolonged and intense symptoms	83%
	6.2 The treatment of migraine should be managed only by a neurologist and/or headache specialist	58%

7. Considering an adult patient undergoing contraceptive therapy: Indicate the level of agreement with the following statement		
		Agreement
In the management of migraine in adult women requiring contraceptive therapy	7.1 The presence of migraine and aura should be carefully considered	100%
	7.2 The choice of contraceptive type is based on the presence of risk factors for ischemia	92%
	7.3 A consultation between a gynecologist and a neurologist/headache specialist is necessary to confirm the diagnosis of migraine with aura	83%
	7.4 The actual thrombotic risk in each patient should be routinely assessed (e.g., screening for thrombophilia) before choosing a contraceptive	83%

8. Considering an adult patient who is pregnant or breastfeeding: Indicate the level of agreement with the following statement		
		Agreement
In the management of migraine in adult women during pregnancy or breastfeeding	8.1 The presence of migraine should be noted during the initial gynecological visit	100%
	8.2 Special attention should be paid to the onset of new-onset migraine during pregnancy	100%
	8.3 The anti-migraine medications considered safe for both the mother and the fetus can always be administered	100%
	8.4 Consultation with a neurologist and/or headache specialist is necessary for the treatment of migraine	92%
	8.5 Acupuncture can be suggested as a therapy to be carried out either as a replacement or in combination with pharmacological treatment	100%

9. Considering an adult patient undergoing assisted reproduction: Indicate the level of agreement with the following statement		
		Agreement
In the management of migraine in adult women undergoing assisted reproduction	9.1 The presence of migraines should be carefully considered before undergoing assisted reproduction techniques	100%
	9.2 The gynecologist should consider the administration of alternative (lighter) hormonal stimulation protocols to reduce migraine pain	100%
	9.3 A consultation with a neurologist and/or headache specialist is necessary for the treatment of migraine	92%
	9.4 Psychological support is recommended	92%

10. Considering a patient in menopause: Indicate the level of agreement with the following statement		
		Agreement
In the management of migraine in menopausal women	10.1 Migraine represents a contraindication to HRT	50%
	10.2 The peri-menopausal phase should be carefully monitored to avoid strong hormonal fluctuations that trigger migraine attacks	100%
	10.3 The onset of newly emerging migraine during menopause/HRT should be carefully monitored	100%
	10.4 Consultation with a neurologist and/or headache specialist is necessary for the treatment of migraine	92%

11. Considering an adult patient undergoing oncological treatment: Indicate the level of agreement with the following statement		
		Agreement
In the management of migraine in adult women undergoing oncological treatment	11.1 The worsening of migraine symptoms during treatment should be monitored	100%
	11.2 Active collaboration between the oncologist and neurologist/headache specialist is necessary	92%

12. General considerations on follow-up management: Indicate the level of agreement with the following statement		
		Agreement
The management of migraines in the various stages of a woman’s life	12.1 Must ensure the continuity of care (follow-up visits, renewal of treatment plans)	100%
	12.2 Must take place in headache centers (hospital-based or university-affiliated) or headache clinics	75%
	12.3 Must take place at the local or community level	92%
	12.4 Must be shared between headache centers (hospital-based or university-affiliated) or headache clinics, and the local community	92%
	12.5 Must involve the role of the caregiver	58%
	12.6 Must include the use of a dedicated digital platform to enhance the exchange of information and documents about patients among various medical professionals	100%
	12.7 Must include the support of telemedicine	100%

13. Training and education of the pharmacist: Indicate the level of agreement with the following statement		
		Agreement
Training and updating on the management of migraine patients for the pharmacist	13.1 Are necessary	100%
	13.2 Must be mandatory	92%
	13.3 Must include instructions for the use of tools (e.g., ID-migraine) to be used for categorizing headaches (migraine or other types)	100%

14. Training and education of the general practitioner and pediatrician: Indicate the level of agreement with the following statement		
		Agreement
Training and updating on the management of migraine patients for the general practitioner and pediatrician	14.1 Are necessary	100%
	14.2 Must be mandatory	100%
	14.3 Must include instructions for the use of tools (e.g., ID-migraine) to be used for categorizing headaches (migraine or other types)	100%

15. Training and education of the specialized physician: Indicate the level of agreement with the following statement		
		Agreement
Training and updates on the management of migraine patients for other specialists (neurologists, gynecologists, oncologists, etc.)	15.1 Are necessary	100%
	15.2 Must be mandatory	100%
	15.3 Must include instructions for the use of tools (e.g., ID-migraine) to be used for categorizing headaches (migraine or other types)	92%
	15.4 Must be implemented given the lack of scientific evidence supporting the optimal management of patients with migraines in specific settings (assisted reproduction, patients with migraine undergoing oncological treatment)	92%

16. Awareness of the condition for patients: Indicate the level of agreement with the following statement		
		Agreement
Among the initiatives to promote awareness of migraine in women, the following should be encouraged	16.1 The use of social media platforms	92%
	16.2 The number of ‘headache awareness days’ throughout the year	92%
	16.3 The availability of questionnaires in pharmacies (e.g., ID-migraine)	100%
	16.4 All of the abovementioned, as the current ones in place are insufficient	100%

16. Awareness of the condition for patients: Please answer the following questions					
	Yes		No		
16.5 Would you consider useful to have a pamphlet for women that highlights the importance of the symptoms and the phases of the patient’s journey	92%		8%		
	Migraine as a stigma	The neglect of the symptom	Reference medical figures	Socio-economic disease burden	Other
16.6 In your opinion, which awareness themes regarding migraine pathology in women should be further explored? (possible multiple answers)	50%	92%	83%	75%	0%

### 3.3 Delphi second round

The four statements that did not reach the agreement during the first round were revised by the steering committee and re-submitted to the panellists’ evaluation during a second round. The full text of the statements/questions and the relative results are reported in Table 2.

**Table 2:** Second round survey full text and results.

1. Awareness of the condition for patients	
1.1 In your experience, the woman suffering from migraine considers migraine (Please rank the following)	
	Most frequent answer
A pathology	3 (50%)
A symptom of another cause (e.g., hormonal fluctuations, cervical issues, etc.)	1 (50%)
A recurring normal and non-pathological condition	2 (50%)

6. Considering an adult patient with menstrual migraine	
6.2 In the management of adult patients with menstrual or menstruation-related migraine: The treatment of migraine should be overseen by (Please rank the following)	
	Most frequent answer
Neurologist/headache specialist	1 (75%)
Gynecologist	2 (42%)
General practitioner	3 (50%)

10. Considering a patient in menopause: Indicate the level of agreement with the following statement		
		Agreement
In the management of migraine in menopausal women	10.1 The type of hormone replacement therapy should be chosen based on the type of migraine and after careful clinical assessment of the patient	75%

12. General considerations on follow-up management: Indicate the level of agreement with the following statement		
		Agreement
The management of migraine in various stages of a woman’s life should include	12.5 The role of a caregiver for patients in whom migraine has a debilitating impact (e.g., limiting the normal performance of daily activities)	75%

### 3.4 General framework, perception of the illness

Overall, migraine is mainly considered as a recurrent non-pathological symptom (option ranking first position for 55% of panellists) rather than a pathology (27%) by women, and even when recognized as a pathology, there is no clear indication for migraine patients about which healthcare professional to contact, according to 83% of the experts (Table 1 statement 1–3 and Table 2 question 1). Among the various professionals that could be consulted, the GP, the pediatrician and the pharmacist emerged as the first figures sought out for general assistance by women with migraine (between 50 and 75% for GP, 92% for pediatricians, and 33% for pharmacist, Table 1, question 3). Importantly, the disease comes with a great burden for patients, both in term of work/economic loss and social aspects (negative impact on social activities, relationship, and overall quality of life) (83% of panellists). The symptoms are perceived as a stigma by the woman in the opinion of 92% of the experts. It takes between 1 and 3 years to get the first medical assessment (for 33% of the panel) and to reach a diagnosis (42%). Less time is needed when a specialized neurological visit is requested (between 1 and 6 months for 50% of the experts) (Table 1, question 2).

The current patient journey is unanimously recognized as non-standardized, without medical references at the territorial level and lacking multidisciplinary interventions. Overall, the need for active collaboration between the neurologist/headache specialist with the different professionals (gynecologist, pediatrician, oncologist) is highlighted.

### 3.5 Management of pediatric and adolescent patients

Currently, the pediatrician is the first healthcare professional dealing with the pediatric patient in the opinion of 92% of the participants, and it is suggested to be the referral figure also in the optimal path according to 83% of panel. The GP is recognized as the first reference for adolescent patients (75% of responders); panellists confirmed the primary role of the GP in this patient subset when considering the desirable patient management (75% of responders). In a young migraine patient, social aspects that might trigger attacks should be evaluated with the involvement of a psychiatrist only if a psychopathological disorder is hypothesized (100%). A need for better awareness of migraine and its implication among parents is necessary according to all the panellists (Table 1, statements 3–5).

### 3.6 Management of women with menstrual migraine

The adult patients experiencing migraine during menstruations usually refers firstly to the gynecologist (92% of the responders). However, the multidisciplinary assessment involving both the gynecologist and the neurologist/headache specialist is considered the optimal approach (67% each). Indeed, specialized consultation is required only in case of prolonged and intense symptoms (83% agreement) (Table 1, statements 3–4 and 6).

### 3.7 Management of women undergoing contraceptive therapy

All the panellists believe that the gynecologist is the healthcare professional firstly seen by the patient with migraine that is on contraceptive treatment, and is also the ideal specialist to refer to. The presence of migraine and aura should be carefully considered in women on contraceptive therapy for all the experts. Ischemia and thrombotic risks should be evaluated for the choice of the contraceptive (92 and 83% of agreement, respectively), and a consultation with the neurologist/headache specialist is required for aura confirmation (83% agreement) (Table 1, statements 3–4 and 7).

### 3.8 Management of women during pregnancy and breastfeeding or undergoing assisted reproduction

The gynecologist is the healthcare professional who the patient refers to and this would also be the ideal reference. A unanimous consensus is reached for a careful evaluation of migraine onset during pregnancy, start of breastfeeding, and before starting assisted reproduction techniques. All the panel agrees on the administration of safe anti-migraine medications during pregnancy and breastfeeding, and in addition, acupuncture can be suggested. For the patient undergoing assisted reproduction, all the experts agree that the gynecologist should consider the administration of lighter hormonal stimulation protocols to reduce migraine pain, even if there are no sufficient data in the literature (Table 1, statements 3–4 and 8–9).

### 3.9 Management of women in menopause

Currently, a woman in menopause suffering from migraine sees the gynecologist for 50% of the responders, but in an ideal situation both the gynecologist (83%) and the neurologist/headache specialist (67%) should be the reference healthcare professional. All the experts agree that the onset of emerging migraine during menopause/hormone replacement therapy (HRT) should be carefully monitored as well as during the peri-menopausal phase, to avoid strong hormonal fluctuations that can trigger migraine attacks. In addition, the type of HRT should be chosen according to the type of migraine only after careful clinical assessment of the patient. The type of HRT therapy and the presence of a caregiver must be considered in each specific situation, according to the status and needs of the woman with migraine (Table 1, statements 3–4 and 9; Table 2, statement 10).

### 3.10 Management of oncological patients

The oncologist is the healthcare professional to refer to in case of migraine during oncological treatment for 92% of the experts, and indeed it is the one who is currently seen by the patients. Monitoring of worsening of migraine symptoms during oncological therapy is required according to the opinion of all the experts. An active collaboration between oncologists and neurologists/headache specialists is also deemed necessary by 92% of the experts (Table 1, statements 3–4 and 11).

### 3.11 General consideration on follow-up, training of healthcare professionals, and patient awareness

The preservation of continuity in patient care emerges as the paramount feature underscored unanimously by the experts. The patient should be assisted either locally or in collaboration with a specialized headache center (92% agreement each). An active role of the caregiver is required only in case migraine has a debilitating impact on the life of the patient (73% agreement). The use of digital tools and telemedicine is suggested to support both patients and healthcare specialists in the opinion of all the experts. Overall, a need for an increased and mandatory education for all the healthcare professionals potentially involved in the management of the women with migraine (including pharmacists, GPs, pediatricians and specialized physicians) is highlighted by the panel. Particular attention should be paid to the use of tools like the ID-migraine questionnaire to identify and classify the type of migraine. There is a good level of patient awareness according to expert opinion, as 92% agree that the woman considers migraine among the possible cause of her symptoms. Nevertheless, efforts must focus on activities that give the right importance to the recognition of the symptoms (92% agreement) and the definition of the healthcare professional to be contacted (83% agreement) (Table 1, statements 12–16).

## 4 Discussion

Real-world evidence studies have shown that people suffering from migraine significantly underestimate the severity of their symptoms and have poor knowledge about the appropriate medical care (26, 29–31). Female patients appear to be mostly impacted; as such, migraine is recognized to be predominantly a female disorder (13–15, 17–19, 37). In this Delphi consensus, the current diagnostic-therapeutic care options for women with migraine of different age and/or with specific health conditions have been investigated. The consensus was reached for most statements at the first round with only four statements re-administered to the panel in a second round after re-phrasing.

Preliminary questions were used to gain knowledge about patients’ perception of the disease, the length of the current diagnostic path in Italy, and the general management of the condition. In agreement with previous studies, women suffering from migraine complain of a high burden (17–19) and are particularly impacted by the stigma affecting their personal wellbeing (38, 39). Nonetheless, experts believe that migraine symptoms are mostly misinterpreted by the affected women and not considered related to an actual disease; as a result, women with migraine may take a long time before actively seeking medical support. In light of these considerations, experts welcome any type of initiative to promote awareness of migraine in women, with the two most relevant topics being “the neglect of symptoms” (92%) and “reference medical figures” (83%). These results are in line with the wide under-recognition of migraine (40) and emphasize the need for an in-depth education and sensibilization of women. The distribution of an informative pamphlet specifically designed for women, focusing on key migraine symptoms and the various stages of the patient’s life, has been endorsed by the panellists. Raising public awareness and education about the disease are crucial points to empower patients, minimize stigma, provide proper medical care, and accelerate the diagnosis (41). Increased awareness would also help to identify specific types of migraine patients, such as those experiencing typical aura without headache (2). This condition may present with mild or even with no headache, and while being commonly associated with visual disturbances, can also manifest other disabling associated symptoms. Such cases should be referred to a headache specialist or neurologist for thorough evaluation and patient education.

The panel has also recognized the need for ongoing training and educational initiatives for pharmacists, GPs and specialized physicians. As previously pointed out, pharmacists often serve as the initial point of contact for people with migraine who seek assistance in managing acute and prolonged pain (19, 42, 43). Moreover, pharmacists are also the first contact of the significant population of women that suffer from occasional headaches, with mild to moderate, or even severe attacks of frequency of around one per month. As such, their formation is essential to gain knowledge on headache disorders and assist women with migraine by suggesting suitable treatments, including over-the-counter medicines for controlling sporadic attacks, encouraging adherence to therapy, and advising on GP or specialist consultation (19, 42, 43).

### 4.1 Migraine in pediatric age

Migraine attacks may have an early onset, frequently occurring before adulthood (44); thus, special attention should be directed toward the pediatric population to ensure proper diagnosis and intervention. When discussing migraine management in children and adolescent girls, experts stress that patients of this age should be carefully assessed. Although there are national and international guidelines to guide the diagnosis and treatment of headaches in pediatric patients (45, 46), the critical transition between pre-puberty and post-puberty is not sufficiently emphasized. Specific attention should be given to the social aspects of adolescents in their everyday life, identifying situations and experiences that represent a source of anxiety and are associated with migraine [i.e., parental depression, antisocial behavior and alcoholism (47), living in single parent or patchwork families (48)]. The identification of socio-environmental factors that trigger migraine attacks is considered the most appropriate approach in pediatric and adolescent girls. This allows for planning a socio-behavioral intervention, rather than psychotherapeutic procedures, aimed at overcoming or avoiding unpleasant situations. In addition, not all the migraine medications available for adults can be used in pediatric patients (46, 49). The panel agrees that, if pediatric migraine does not improve with the sole socio-behavioral intervention, this should be combined with a pharmacological treatment.

### 4.2 Menstrual-related migraine

Among women of childbearing age who suffer from migraine, up to 22% experience menstrual migraine (50). Typically, management of this migraine subtype involves employing the same acute and preventive therapies recommended for other types of migraines (51), as previous studies demonstrated their efficacy (52). According to the experts’ panel, patients with menstrual migraine should be routinely followed by GPs and they should seek a neurologic specialist consultation only in case of prolonged and intense symptoms. Given that the panel agrees on the key role played by the gynecologist for patients of this age, a coordinated intervention between the neurologist/headache specialist and the gynecologist is encouraged to suggest specific medications and perimenstrual prophylactic treatments.

### 4.3 Adult women undergoing contraceptive therapies

In the case of adult women undergoing contraceptive therapy, the presence and the type of migraine should be carefully evaluated by the prescribing clinician. According to the World Health Organization guidelines, migraine with aura represents an absolute contraindication to the use of estrogen-progestin hormonal contraceptives (combined hormonal contraceptive, CHC) (53), as it is associated with increased risk of cerebrovascular ischemic events (54, 55). Migraine with aura also appears to be associated with increased risk of cardiovascular events (myocardial infarction, angina, death due to cardiovascular disease) (54). Even if migraine without aura is compatible with the use of CHC (56), any new persisting headache or increased headache frequency/intensity occurring during the contraceptive treatment should be thoroughly investigated (57). Progestin-only contraceptives are reckoned safer than CHC (58); as such, they should be used instead of CHC in women experiencing migraine with aura (59). The use of progestin-only contraceptives is also preferred in women with migraine without aura in presence of additional risk factors (e.g., smoking, hypertension, age > 35 years old) (57, 59). Notably, smoking habit is common among subjects with migraine and may increase the risk of ischemic stroke (60), especially in women under the age of 45 who use oral contraceptives (61, 62). According to the experts’ experience, most women receive CHC without being asked about the presence of migraine symptoms. The panellists stress that the proper recognition of migraine, and especially aura, is imperative; for this reason, the neurologist/headache specialist should be routinely involved to ascertain the presence of aura before prescription of CHC. An in-depth evaluation of the actual thrombotic risk should be carried out in selected patients to define the most appropriate contraceptive method.

### 4.4 Adult women: pregnancy and breastfeeding

The experts affirm that pregnant and breastfeeding women should receive special care if they have migraines, particularly if they develop migraine during pregnancy. Migraine increases the risk of gestational complications, including hypertension, preeclampsia, as well as vascular complications, such as ischemia (63). The fear for adverse events upon prescription of anti-migraine medications should be overcome by the knowledge of medications now considered safe, which can be administered during pregnancy or breastfeeding (64). The effectiveness and safety of acupuncture as an alternative therapy for migraine during pregnancy is emphasized (65); the experts agree that patients should receive acupuncture either alone or in combination with pharmacological treatment. The current limitation to the use of acupuncture is attributed to the lack of public resources and specialized personnel that can guarantee this technique within the public Italian National Healthcare System. It is generally agreed that the neurologist/headache specialist should be implemented within the diagnostic-therapeutic path for pregnant and breastfeeding women.

### 4.5 Adult women undergoing assisted reproductive procedures

The experts highlight the absolute lack of guidelines and literature related to women with migraine who receive hormonal treatments in the context of assisted reproduction. Like women receiving contraceptive methods, this subset of patients should be carefully examined for the presence of migraine before undergoing reproductive techniques. For this reason, a multidisciplinary team including neurologists/headache specialists is deemed necessary. In addition, even in the absence of clinical trials on this topic, the use of alternative hormonal stimulation protocols as well as a psychological support should be prioritized, as many patients abandon assisted reproduction attempts due to the exacerbation of migraine symptoms associated with hormonal stimulation. Clinical trials on women undergoing assisted reproductive procedures are mandatory.

### 4.6 Women in menopause

Despite migraine tends to decrease with age (13, 14), late premenopausal and perimenopausal periods are associated with the highest risk of migraine, due to the hormonal variation typically observed in these stages (66). While perimenopausal hormonal fluctuation should be avoided as much as possible to prevent migraine attacks (67), it is well-known that HRT is associated with increased risk of cardiovascular and cerebrovascular ischemic events (68). While HRT can be administered in women with migraine (66, 67, 69), experts agree that the onset of newly emerging migraines during menopause, and especially during HRT, should be carefully investigated. To lower the risk of adverse events, gynecologists should choose the type of HRT case-by-case, according to the specific features of the patient; non-estrogen compounds, phytoestrogens or physiological doses of natural estrogen should be considered valid alternatives among the menopause therapeutic options (51, 67). The neurologist/headache specialist should be consulted to decide on the best anti-migraine treatment. Given the scarcity of literature data about the optimal management of migraines in menopausal women, panel members suggest sharing existing experiences to enhance collaboration among specialists, their awareness, and preparedness.

### 4.7 Women undergoing oncological treatments

The panel also discussed the management of migraine in women undergoing oncological treatment. Some oncological therapies are associated with worsening migraines, especially hormonal treatments for breast cancers, that significantly impact on the quality of life (70). The ideal pathway for a cancer patient with migraines should include a direct and active collaboration between oncologist and neurologist/migraine specialist. This organization would allow for the immediate referral of patients by the oncologists to the neurologist/headache specialist when migraines worsen. The experts emphasize that the treatment of migraine in oncologic patients is extremely difficult due to lack of literature.

## 5 Conclusion

Considering all the evidence presented, the current Italian management of migraine in female patients requires profound improvements to guarantee an optimal, standardized, and continuous care in every phase of women’s lives. Along with the urgency of increase migraine awareness among women, the experts highlight the need for: (1) increased multidisciplinary (intended as a close collaboration between the neurologist/headache specialist and any other medical professionals possibly involved in migraine care throughout women’s lives); (2) increased collaboration between headache clinics and local/community healthcare services; and (3) increased education about migraine management and diagnostic tools via periodic updates (dedicated to all the professional stakeholders potentially supporting women with migraine, including pharmacists, GPs, pediatricians, and other specialists). The use of telemedicine and the development of digital platforms to exchange patient information are encouraged to speed up the diagnostic-therapeutic process and facilitate multidisciplinary collaboration. Experts confirm that the role of the caregiver is required only to ensure the normal accomplishment of everyday activities in cases where women suffer from debilitating migraines.

The low number of experts included in this study represents its main limitation. Moreover, the panellists’ responses were often based on subjective opinions and experiences rather than being objectively assessed, due to the lack of data and guidelines in the literature. Nevertheless, the solid real-world experience of the panellists is a major strength of the study. Despite being known and accepted that migraine mostly affects females, to our knowledge, this is the first Delphi study that delves into the current management of this specific subset of patients with migraine according to their age and conditions within the Italian healthcare system. In addition, the panel included professionals with a broad range of different expertise to capture multiple points of view and substantiate the consensus.

This Delphi comprehensively addressed female migraine by advocating the need and finding solutions to increase awareness and appropriate care; thus, treatment choices and management strategies were not discussed. The panellists did not examine other relevant topics, such as the potential benefits of cannabis use for migraine and its impact on medication overuse headache (71). While these aspects were not considered specifically related to the female condition, their exclusion represents a limitation of this study.

In conclusion, this study offers an overview of migraine management in women through the consensus reached from a panel of experts in the field. The findings offer a strong foundation to improve the diagnostic-therapeutic approach for female migraine patients by addressing their specific age and health-related needs. Finally, the literature overview highlighted an impressive lack of scientific evidence in many aspects concerning the needs of women suffering from migraine that should be addressed.

## Data availability statement

The original contributions presented in the study are included in the article/supplementary material, further inquiries can be directed to the corresponding author.

## Ethics statement

Ethical review and approval was not required for the study on human participants in accordance with the local legislation and institutional requirements. Written informed consent from the [patients/participants OR patients/participants legal guardian/next of kin] was not required to participate in this study in accordance with the national legislation and the institutional requirements.

## Author contributions

SC: Conceptualization, Investigation, Methodology, Writing – original draft, Writing – review & editing. PBa: Conceptualization, Investigation, Methodology, Writing – original draft, Writing – review & editing. CF: Conceptualization, Investigation, Methodology, Writing – original draft, Writing – review & editing. LB: Methodology, Writing – original draft, Writing – review & editing. PM: Methodology, Writing – original draft, Writing – review & editing. NO: Conceptualization, Investigation, Writing – original draft, Writing – review & editing. SB: Investigation, Writing – original draft, Writing – review & editing. PBr: Investigation, Writing – original draft, Writing – review & editing. DC: Investigation, Writing – original draft, Writing – review & editing. LM: Investigation, Writing – original draft, Writing – review & editing. SM: Investigation, Writing – original draft, Writing – review & editing. RS: Investigation, Writing – original draft, Writing – review & editing. FF: Conceptualization, Investigation, Methodology, Writing – original draft, Writing – review & editing. GA: Conceptualization, Investigation, Methodology, Writing – original draft, Writing – review & editing.
